# Aortic pulse wave velocity in obesity as assessed by magnetic resonance imaging

**DOI:** 10.1186/1532-429X-11-S1-P26

**Published:** 2009-01-28

**Authors:** Oliver J Rider, Upasana Tayal, Jane M Francis, Monique Robinson, James P Byrne, Kieran Clarke, Stefan Neubauer

**Affiliations:** 1 University of Oxford, Oxford, UK; 2 Southampton General Hospital, Southampton, UK

**Keywords:** Obesity, Abdominal Aorta, Pulse Wave Velocity, Excess Weight Loss, Aortic Stiffness

## Objective

Our aim was to investigate the effects of obesity and weight loss on aortic Pulse Wave Velocity as assessed by vascular magnetic resonance imaging.

## Introduction

Obesity is an escalating global health problem associated with both an increased risk of death and an increased risk of cardiovascular events. The various mechanisms by which obesity mediates cardiovascular risk are not well understood but may be related to aortic stiffness, both present in obesity and linked to increased mortality. Our goal was to determine the effect of obesity, in the absence of the traditional cardiovascular risk factors on aortic pulse wave velocity (PWV) as assessed by vascular magnetic resonance imaging, a reliable, reproducible and accurate clinical measure of aortic stiffness.

## Methods

Fifty obese (BMI 38.3 ± 6.8 kg/m^2^) and eighteen normal weight controls (BMI 22.0 ± 1.7 kg/m^2^) with no identifiable cardiovascular risk factors underwent vascular magnetic resonance imaging at 1.5 T to assess of PWV at three points, the ascending (Ao) and proximal descending aorta (PDA) at the level of the pulmonary artery and the abdominal aorta (AA) 12 cm below the pulmonary artery. Aortic flow/time curves, at these three points, were used to calculate the arrival times of aortic pulse waveform. The time of arrival of the pulse wave (Δt (ms)) was determined to be the intercept of the tangent to the curve at the half maximal point of flow. The distance over which the pulse wave had travelled (Δx) was measured from oblique sagittal aortic images. PWV was then calculated as Δx/Δt. 28 subjects underwent repeat imaging after weight loss, 16 obese subjects underwent a supervised low glycaemic index diet, while twelve underwent bariatric surgery.

## Results

Obese subjects and normal weight controls were well matched for age, height, systolic blood pressure, fasting plasma glucose and total serum cholesterol. concentration compared to controls (p < 0.005). Obesity was associated with a 14% increase in PWV when compared to the normal weight controls (p = 0.021) and furthermore was associated with significantly higher leptin (p < 0.001) and C-reactive protein levels (p < 0.01), factors known to affect aortic mechanical function. After a one year period of weight loss (Average 21 kg, 50% of excess weight loss) there was a significant 14% decrease in aortic pulse wave velocity when measured between the ascending and abdominal aorta (p = 0.03). In contrast, while showing a trend, aortic pulse wave velocity improvements in proximal sections did not reach statistical significance. Both leptin and C-reactive protein levels were also reduced with weight loss. See Table 1.

**Table 1:** Table 1

	Control (n = 18)	Obese (n = 50)	p value
PWV: Aortic Arch (m/s)	9.80 ± 8.26	9.86 ± 6.26	0.34
PWV: Descending Aorta (m/s)	4.34 ± 0.82	5.52 ± 6.12	0.25
PWV: Ascending Aorta to Abdominal Aorta (m/s)	5.0 ± 0.6	5.7 ± 1.5	0.02
	**Obese Pre Weight Loss** **(N = 28)**	**Obese Post Weight Loss (N = 28)**	**p value**
PWV: Aortic Arch (m/s)	10.3 ± 7.2	10.1 ± 7.7	0.92
PWV: Descending Aorta (m/s)	6.2 ± 8.3	4.3 ± 1.4	0.25
PWV: Ascending Aorta to Abdominal Aorta (m/s)	5.8 ± 2.0	5.0 ± 1.0	0.03

## Conclusion

Obesity, in the absence of the traditional risk factors of hypertension, diabetes and hypercholesterolaemia, is associated with significant increases in aortic PWV, a non invasive clinical measure of aortic stiffness known to be independently predictive of cardiovascular mortality. Furthermore, a period of one year of weight loss is associated with a reduction in pulse wave velocity. This may provide a potential explanation for both the link between obesity and increased mortality, and the link between weight loss and increased survival.

